# Nonspecific hebbian neural network model predicts musical scales discreteness and just intonation without using octave-equivalency mapping

**DOI:** 10.1038/s41598-022-12922-x

**Published:** 2022-05-25

**Authors:** Toso Pankovski, Ana Pankovska

**Affiliations:** Montreal, Canada

**Keywords:** Biophysical models, Auditory system, Perception, Sensory processing

## Abstract

This study continues investigating the consonance-pattern emerging neural network model introduced in our previous publication, specifically to test if it will reproduce the results using 100-fold finer precision of 1/100th of a semitone (1 cent). The model is a simplistic feed-forward generic Hebbian-learning generic neural network trained with multiple-harmonic complex sounds from the full auditory sound spectrum of 10 octaves. We use the synaptic weights between the neural correlates of each two-tone from the said spectrum to measure the model’s preference to their inter-tonal interval (12,000^2^ intervals), considering familiarity as a consonance predictor. We analyze all the 12,000 intervals of a selected tone (the tonic), and the results reveal three distinct yet related features. Firstly, Helmholtz’s list of consonant intervals re-emerges from the synaptic weights of the model, although with disordered dissonant intervals. Additionally, the results show a high preference to a small number of selected intervals, mapping the virtually continual input sound spectrum to a discrete set of intervals. Finally, the model's most preferred (most consonant) intervals are from the Just Intonation scales. The model does not need to use cross-octave interval mapping due to octave equivalence to produce the said results.

## Introduction

In this study, we focus on processes involved in predicting the following three important features of tonality: discreteness of tonal intervals in musical scales, Just Intonation, and familiarity as a predictor of consonance—the consonance order of intervals. Immediately at the beginning, we need to prevent possible misunderstanding—the ambiguities of natural languages introduce slight confusions when reviewing the results of the Science of Music in the context of finding bio-physical systems that yield listeners’ musical preferences. The musical science literature uses words such as “methods of creation of scales” when referring to mathematical methods of producing a theoretical/instructional list of intervals, rather than denoting attempts of finding the processes of emergence of intervals’ tangible traces in the bio-physical systems. In this paper, we attempt to make a distinction between the “regular” mathematical methods of describing the correlations between the tonal systems and the frequency ratios of the involved tonal intervals used by others in the science of music and tonality, and our objective of analyzing a model based on real-life bio-physical systems that produce tangible (physical) traces of the results that directly maps to the tonal intervals of scales. Since this study combines the disciplines of tonality and neural networks, we provide a short introduction to most of the involved concepts.

Frequency is one of the main properties of sound, which in turn, Scnupp et al.^1^ defines it as a pressure wave that propagates through the air. Frequency is defined as the number of oscillations per unit of time, and its nature is continuous, meaning a tone (which is a sound of a defined frequency) can have a frequency of any positive real number. Furthermore, there are infinite possible choices within any range of real numbers; therefore, the tonal/sound spectrum is continuous.

Tonality, in music, is a principle of organizing musical compositions around a selected note, named *tonic*. In contrast to frequency’s continual nature, the vast majority of *tonal systems* across diverse cultures are used to produce tonal *scales* that use only limited sets of *discrete* tones (more precisely—the intervals between the tones)^2^ (Burns). Thompson^3^ proposes that scales provide a “psychological framework within which music can be perceived, organized, communicated, and remembered”. As per Sachs and Kunst^4^, exceptions of scales’ discrete nature exist, yet they are rare. Furthermore, there is no agreement on why the scales are made of discrete sets of intervals, although the sound spectrum that can be vocalized is continuous. To enforce this apparent mystery, we remind that the borderline tonal interval a human hearing can resolve is approximately 10/100th to 20/100th of the most prevalent smallest of intervals found in the scales—the *semitone* (per Burns^2^ and Bonnard et al.^5^).

Terhardt^6^ proposes that using discrete (and limited by count) tones in tonal systems is biologically beneficial to prevent information overload in species with highly developed sensory systems, such as humans. Bidelman and Heinz^7^, armed with powerful mathematical and statistical tools (but not using biophysical models), while attempting to reproduce the Helmholtz’s^8^ list of (pre-selected) intervals order by consonance, predict the discreteness of the intervals in musical scales within the continuous realm of sound frequencies. In the same paper, the authors suggest that the discrete nature of the intervals in scales are produced by a “central pitch processor”—a term also used by Goldstein^9^ and Terhardt et al.^10^—representing a sub-system of the auditory system that stores internal templates of pitches and recognizes the perceived stimulus by matching its harmonic information to the internal templates. However, they admit that the apparatus of such a “processor” is unknown (a black box)—and consequently the “machinery” of mapping the continual auditory spectrum to a discrete set of intervals.

**Figure 1:**
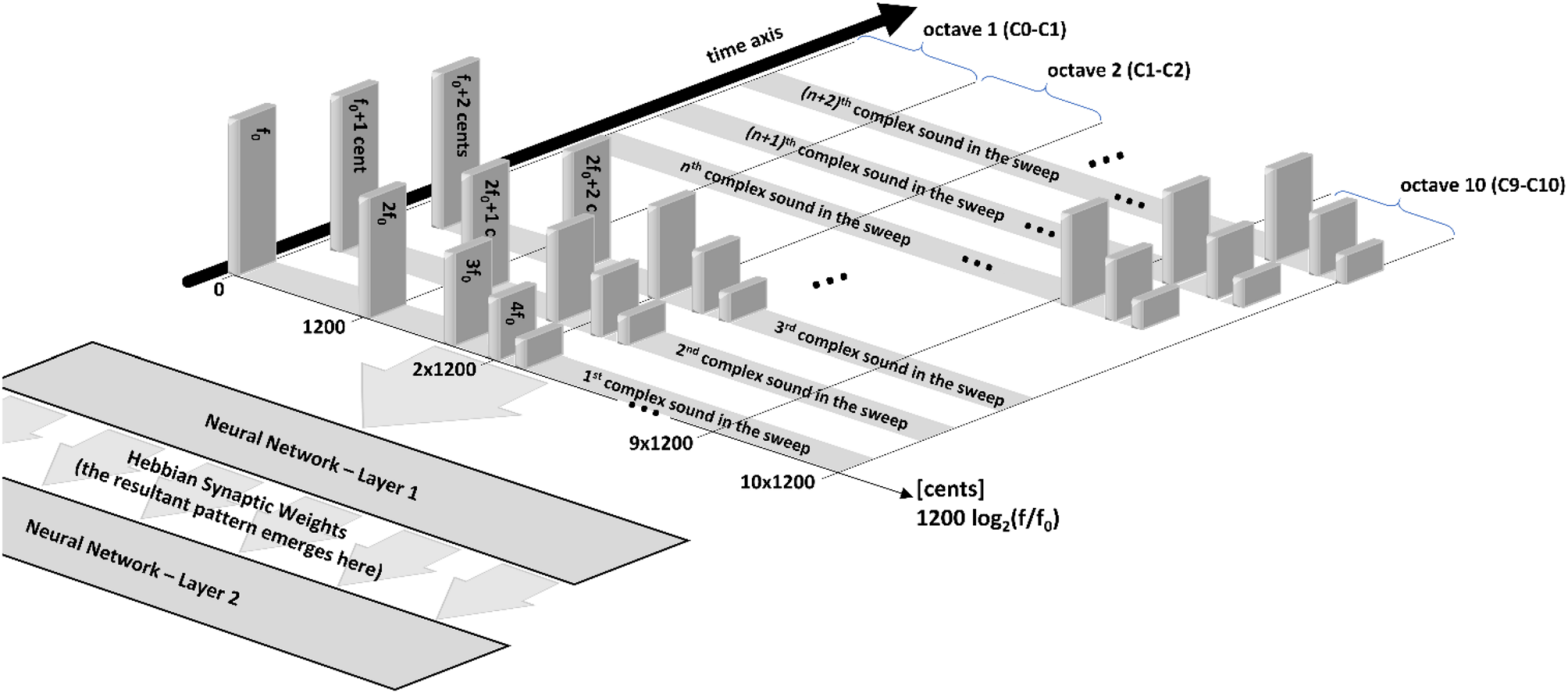
Schematic of the model used in this study. The neural network is fed by a train of complex 5-harmonic sound stimuli, starting with the lowest base tone C0 of f0 = 16.352 Hz (the zero reference of the relative unit of cents), sweeping the whole auditory sound spectrum up to C10, the 12000th sound in the train (16,744.036 Hz, or 12,000 cents), in steps of 1 cent. The diagram implies tonotopy, yet it is not required to produce the results; tonotopy is only helpful to facilitate the analyses. Each neural network layer contains neural correlates (12,000) of each harmonic from the whole input auditory spectrum. The links between layer 1 and layer 2 of the neural network feature Hebbian learning, the most important feature in the model; the results emerge in the 12000^2^ synaptic weights of the said links.

This study is a continuation of our previous work^11^ (Fig. 1), where we suggest a *causal* explanation of the consonance/dissonance phenomena based on the Hebbian learning—placing the “pitch processor” in the brain. In that study, we use an inter-tonal resolution of the inputs of one semitone (100 cents), members of the 12-tonal well-tempered scale—meaning, the spectrum of the sounds is already discrete at the input. In the said paper, the model we use is a simplistic feed-forward generic Hebbian-learning neural network trained with multiple-harmonic sounds. Since the harmonics of the base tone of the sound are not always members of the 12-tone tempered scale, each harmonic is represented (and replaced) by the tone of the well-tempered scale that has the closest frequency to that harmonic, introducing imprecisions in the study. Without getting into all the details of the inner workings of a generic neural network, here we explain just the basic principles that help to understand the model. The neural network consists of neural elements—neurons (abstractions of real neurons), where each neuron correlates to a given tone from the whole input auditory spectrum of 120 tones (so we call them *neural correlates*). Every two neurons are connected with a uni-directional link called a synapse, where the “source” neuron is called pre-synaptic, and the “target” neuron—is *post-synaptic*. The synapse has a weight acting as an amplification modulator of the signal passing through it. When we present a sound stimulus to the neural network, the neurons that correlate to the tones contained in that stimulus become excited. At that moment, the model applies the Hebbian learning paradigm (postulated in its original form by Hebb^12^), which is one of its most simplified forms can be described as “the synaptic weight (amplification) between two neural correlates is increased direct-proportionally to the excitement magnitudes of those neural correlates (in arbitrary units).” This synaptic weight change causes the model to *remember* that those two neural correlates were simultaneously excited at some point in the past. As a consequence, at a future occasion when only one of the neural correlates is excited, the other neural correlate will increase its excitement through the synaptic link with increased weight (amplification). Our previous study shows that if we exclude the Hebbian learning from the model, the neural network will never encounter any incidence of two tones forming an interval beyond the six most consonant (as per Helmholtz). As we explain later, the Hebbian paradigm introduces deviations, becoming the core advantage of our model over the other non-Hebbian-learning studies that attempt similar goals. The said deviations result from the adjusted synaptic weights causing increased excitement to the linked neural correlates. As we see later in the current study results, such deviation is required to yield preference of the model to less-consonant intervals.

In the model of our previous study, we consider the synaptic weights between the neural correlates of each two tones as measures of the model’s preference to their inter-tonal interval, that is—of interval’s consonance; here, we explain why. In McLachlan et al.^13^, suggest that long-term memory plays a role in the perception of consonance phenomenon. Then, Hebb^12^ proposed that the long-term memory patterns are registered within the synaptic weights. In addition, in his EEG (Electroencephalography) study^14^, Janata found a correlation between the P3a component of the P300 ERP (event-related potential) brain waves and the perception of dissonant sounds. Not excluding other possible causes contributing to the consonance/dissonance phenomena, his finding suggests some correlation between the dissonance and the perception of stimuli to which we are not accustomed—others concur (Duncan-Johnson and Donchin^15^, Polich^16^, Proverbio et al.^17^, and especially Pack et al.^18^). Consequently, the consonance could be related to well-established long-term memory patterns (*familiarity*), which is why we are interested in the synaptic weights. In their recent (2021) impressively comprehensive study on Consonance/Dissonance (C/D), Eerola and Lahdelma^19^ suggest that “Today, the research field is starting to reach a consensus that the overall perception of C/D in simultaneous sonorities in the Western musical culture is arguably based on a combination of roughness, harmonicity, and familiarity.” That combination of familiarity (represented by the high-magnitude synaptic weights) and harmonicity play core roles in our model. Our model does not explicitly consider the third concept highlighted by Eerola and Lahdelma—the roughness—demonstrating that it is not required for our simplistic bio-physical model to yield the obtained results. To avoid misunderstanding, while our model does not require roughness to produce its results, we do not propose that it does not affect the Consonance/Dissonance phenomena.

**Figure 2:**
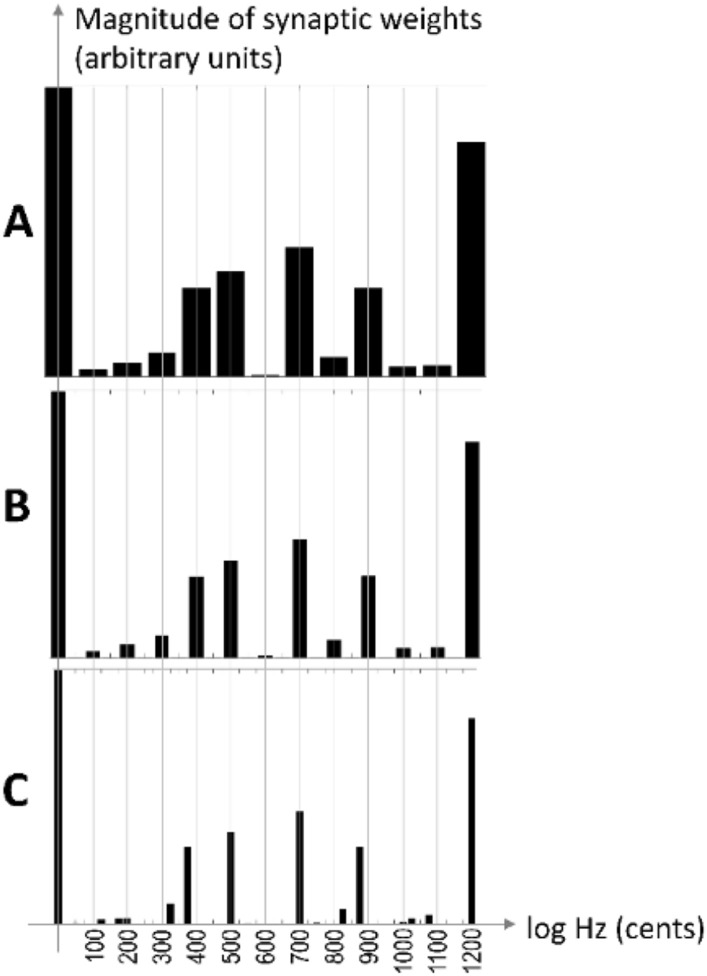
The discreteness and the emerged pattern seem invariant of the input sound spectrum resolution. (**A**) Input sound spectrum resolution of 100 cents. (**B**) Input sound spectrum resolution of 50 cents. (**C**) Input sound spectrum resolution of 25 cents. In C (25 cents), one can observe that additional intervals with much smaller magnitudes emerge. The MATLAB scripts used to produce these graphs are in the Supplemental Information package: (**A**) InputPerSemitone_1.m. (**B**) InputPerSemitone_2.m. (**C**) InputPerSemitone_4.m. A clue from the unpublished results from our previous study^11^.

In our previous publication^11^, we analyze all the 120 intervals of a selected tone (the tonic) from the 12-TET scale, and the Helmholtz’s list of just intonation intervals ordered by consonance is clearly and completely found in the synaptic weights of the neural network (ignoring the cent-differences between the 12-TET and the just intonation scales). Our previous study uses *discrete* input tonal elements with a frequency resolution of 100 cents; without surprise, that model yields discrete results of the same resolution. Interestingly, we obtained additional (unpublished) results during the simulations using an input sound spectrum of finer inter-tonal resolution of 50 and 25 cents. Expectedly, the produced intervals have a span/resolution of 25 and 50 cents, yet, surprisingly, the model kept favoring intervals of approximately 100 cents (Fig. 2). This suggests an interesting clue—the discreteness and the emerged consonance pattern may be invariant on the input sound spectrum’s frequency resolution. Finally, considering Partch’s comment^20^ that famously highlights and condenses the consonance-superiority of Just Intonation and its relations to other tunning systems: “… and the ear does not budge for an instant from its demand for a modicum of consonance in harmonic music nor enjoy being bilked by near-consonances which it is told to hear as consonances. The ear accepts substitutes against its will.” we are interested to see if- and what intervals from the Just Intonation tuning will the model yield as the most consonant.

The clues are the inspiration to continue investigating our previous model in the present study using much-finer frequency resolution of the input sound spectrum. Consequently, we chose a 100-fold finer resolution than the one used in our previous study: the interval span of 1 cent between two adjacent tones of the input spectrum. This gives a total of 12,000 tones spanning 10 octaves in the (virtually continual) sound spectrum of the stimuli; these tones form 12,000^2^ intervals and the same number of synaptic weights (measures of consonance) interconnecting each two tonal neural correlates. Furthermore, the results are analyzed in a context of a selected tone (the tonic) from the mid-range of the auditory spectrum (C4) so that it will be far from the boundary influences that may appear near to the lowest and highest frequency ranges.

Although we do not additionally review the relation between the just intonation and other types of tunning, in order to provide additional musical context, we shortly explain Pythagorean and well-tempered tuning. Pythagorean tuning is a system of musical tuning in which the frequency ratios of all intervals are based on the ratio 3:2 (fifths). Pythagoras constructed a musical scale by “moving successively up and down by fifths” (Thompson^3^). Unfortunately, this method leads to unpure intervals (Pythagorean comma). The equal temperament (well-tempered) “distributes this discrepancy equally”^3^ over the chromatic scale, making it highly popular among Western musicians. We end this short tonality reminder by citing Thomson^3^, suggesting that just intonation optimizes consonance in intervals by tuning “other scale notes such that their fundamental frequencies relate to that of the tonic by small integer ratios”.

As opposed to the choice that our model considers the brain (the Hebbian learning paradigm) a required component in the processes involving pitch processing and, therefore, consonance, Bidelman and Heinz^7^ suggest that the human brain is not required for the models to produce consonance and pitch phenomena. They also suggest that the “central pitch processor” (the said “black box” the processes responsible for the perception of pitch, and consequently consonance/dissonance) is located within the cochlea (outside of the brain) – additionally implying that the Hebbian learning is not part of the process. However, Gockel et al.^21^ offer experimental evidence on humans opposing Bidelman and Heinz’s proposal. Finally, in this context, we mention that Gill and Purves^22^ offer a solid overview of musical scales highlighting their similarity to harmonic series, yet they do not offer a tangible explanation of the processes responsible for their emergence.

In this study, to demonstrate the core benefit of our method over the pure statistical and mathematical models used in studies that do not involve Hebbian learning (and neuroplasticity, in general), we use Terhardt’s old paper^23^. Therhardt’s model, analyzing it from a perspective with a certain level of abstraction, uses a model similar to ours. In both studies—his and our—repeated stimulations of the models with natural sounds (being a base tone with its first few harmonics) sweeping the whole auditory spectrum produce association (synaptic) weights of the intervals that correspond closely to the top-intervals of the Helmholtz’s consonance list. Both models demonstrate a transformation of the virtually continual input sound spectrum to discrete preferred intervals. The crucial difference between the two models is that in Terhardt’s, the subsequent stimulations do not depend on already established associations weights, whereas ours does. Terhardt’s model, being very similar to ours, is investigated here, and for a fair comparison, we again use a frequency resolution of 1 cent. Then we compare both outcomes and discuss the similarities and differences.

### X Hypothesis

By using the simplistic generic Hebbian-learning neural network auditory model of our previous study^11^, refining its input tonal resolution to 1 cent, we expect that the resultant synaptic weights of the intervals’ neural correlates of a selected tone (the tonic) should yield tonal intervals from the Just Intonation tuning—producing a discrete list of most consonant intervals as per^8^. Also, such results should demonstrate the approximate tonal scale discreteness invariance on the used input spectrum resolution, and the inter-tonal scale span *approximately* 100 cents (one semitone).

To clarify, the input tonal interval resolution of 1 cent is a 100-fold refinement from that of the original model—which is the 1/100th (log) fraction of a semitone interval, and the 1/1200th (log) fraction of the octave, thus effectively we could consider the input sound spectrum of the model to a *virtually continuous* one.

## Results

**Figure 3:**
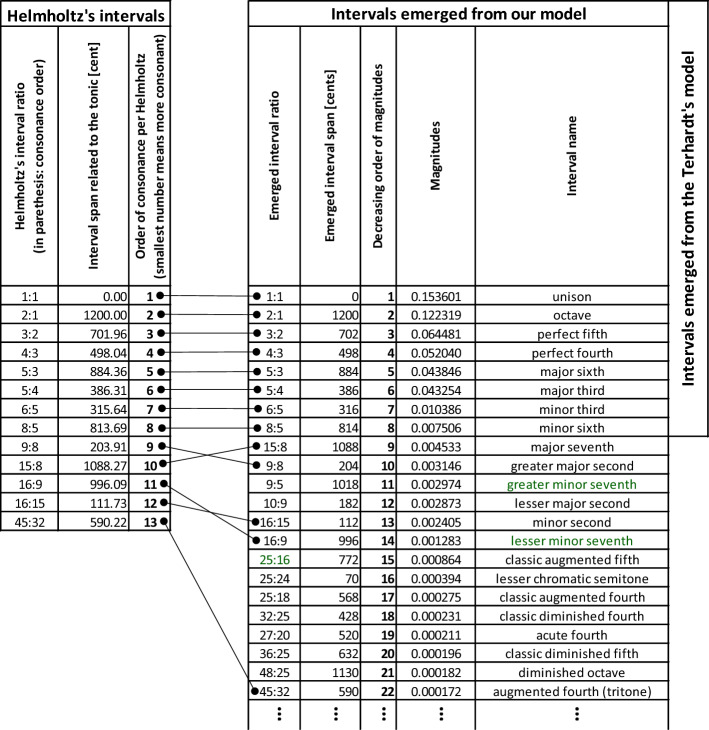
Comparative layout of the obtained results. The list “Helmholtz’s Intervals” contains the 13 intervals used by Helmholtz^8,24^ in their order of consonance reported in those two studies. The list “Intervals Emerged from our model” is the list of intervales that our model yielded, sorted by decreasing order of consonance, i.e., by descending magnitude of their synaptic weights. The six top-most intervals of the right-most list are those yielded by Terhardt’s model. This presentation shows how the top-most consonant intervals yielded by our model match Helmholtz’s consonance order. The “Intervals Emerged from our model” has 601 entries, and by performing additional sweeps, all the possible 1200 intervals from the span of an octave would emerge.

We collected data about various musical scales used across ages and cultures and compared them with the intervals yielded by our model. We also compared the collected data with our version (an abstraction) of Terhadrt’s model^23^. Also, we review separately the 13 intervals ordered by consonance in Helmlohtz^8^ and Lots and Stone^24^ (the left table on Fig. 3).

In the right table of Fig. 3, we enlisted the intervals that emerged from our model, sorted by descending order of their synaptic weights (our selected measure of consonance); this way, we have the intervals yielded by the model as a most consonant, on the top. Note that the right table contains 601 intervals emerged from 8 sweeps of 5-harmonics input sounds; by increasing the number of sweeps, all the 1200 intervals from the octave will emerge (as per the neural network design)—although the rest ~ 600 will have the most negligible magnitudes. From all the emerged, Fig. 3 only shows the first 22 intervals, having the tritone as last, matching the last entry in the left table.

We intended to show the list of collected intervals from literature (total of 1162) in comparison to those that emerged from our model. However, since all the 1200 possible intervals emerge from our model (after a sufficient number of sweeps), and the collected interval list has no particular order, there is no use of showing such a comparison.

The table is only a small extract (the top portion) of the entire table found in the document containing additional results and analyses, located in the Supplementary Information package, under the filename RESULTS.xlsx.

Although the model yields all the possible 1200 intervals (after a sufficient number of sweeps), it is evident that the most consonant emerged intervals (with highest magnitudes of synaptic weights) match the intervals from the Just Intonation 5-limit system. Furthermore, we notice that both major second “versions” (9/8 the greater major second and 10/9 the lesser major second) are yielded among the top 22 most consonant intervals. We report similar findings for the lesser- and the greater minor seventh. On the other hand, we observe that the least consonant intervals do not match the Helmholtz order (the 9th and the 10th orders are swapped) or are pushed down in the consonance order.

The intervals that emerged from Terhardt’s model^23^ are indicated in the most-right column of Fig. 3; we observe that Terhadrt’s model only yields the six most consonant intervals, the *First-Order Partners* (introduced in per our previous study^11^).

The (extended) matching result from Fig. 3 is represented graphically in Fig. 7. This figure shows a bar graph of the magnitudes of the emerged intervals against the interval span dimension. The graph resembles Fig. 2, additionally supporting the suggestion of the approximate invariance of both (a) the discreteness of the scales and (b) the consonance pattern on the used input frequency resolution. The number annotations above the arrows show the order of the emerged interval in the list order by decreasing magnitude (lower numbers represent more consonant intervals). The filled arrows point to the matching Helmholtz’s list intervals as per Fig. 3. The empty arrows point to the intervals that our model yielded as more consonant than 16th or 22nd. The upper grayed area contains the First-Order Partners (highly consonant intervals). The lower grayed area contains the Second-Order Partners (less-consonant intervals); the wide gap between the First-Order Partners and Second-Order Partners magnitudes is observable. The intervals that emerged from Terhardt’s model are all in the zone of the First-Order Partners—that model yielded only six intervals.

**Figure 4:**
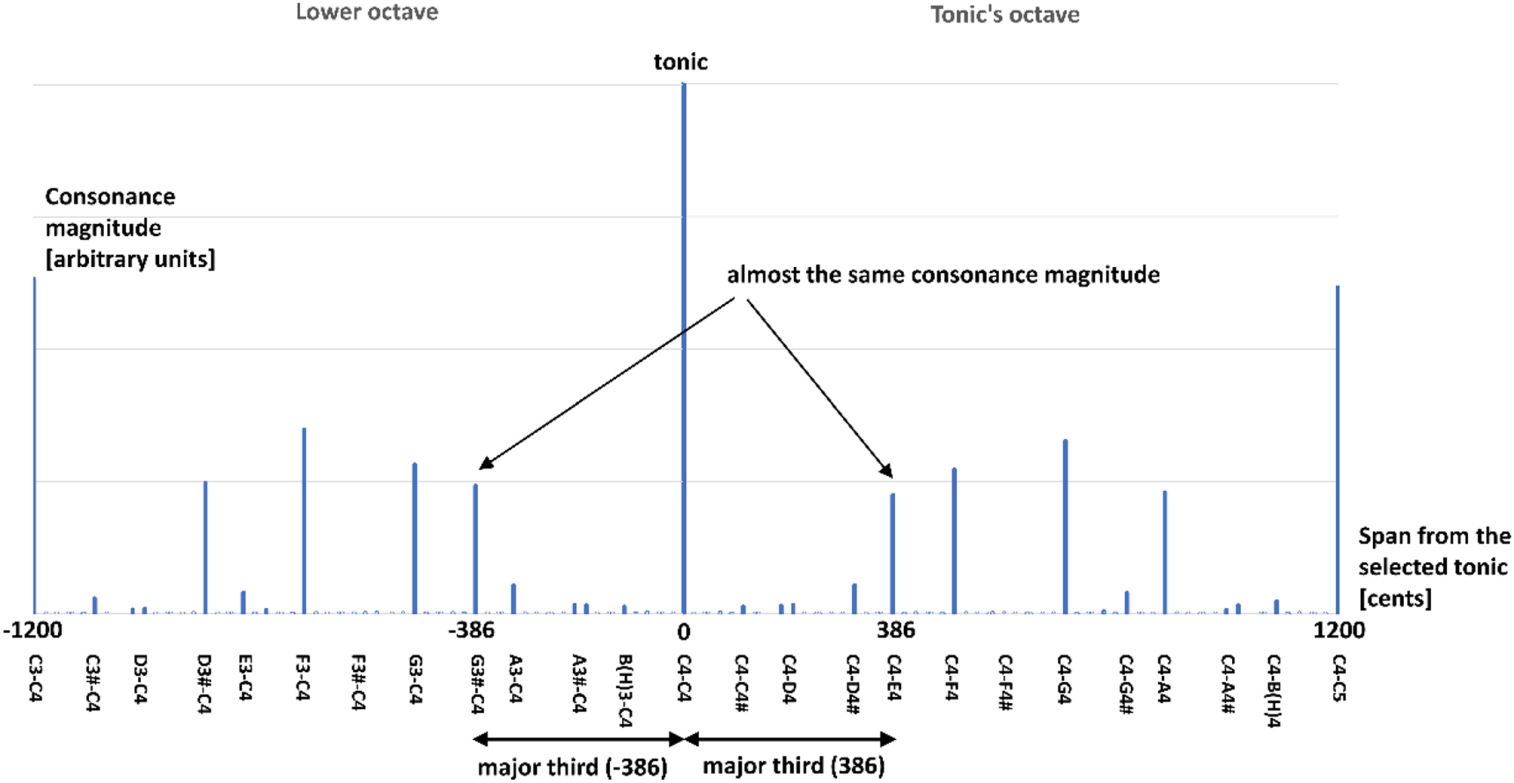
Symetry between the lower- and the tonic’s octave. The figure shows the synaptic weights (the consonance measure) of the emerged intervals, including the selected tonic (C4 in the example shown here) in the octave-span below and above the tonic. The symmetry of consonance between the C3–C4 octave tone (the lower) and the C4–C5 (the upper) is evident. Although the graph shows that the tone G3# forms the minor sixth interval C3–G3# (the minor sixth is an inversion of the major third) may raise appearance about the equivalence of the intervals with their inversion, that is misleading—the intervals’ magnitudes shown on the left portion of the graph is the major third in the key of G3#. The horizontal axis represents the interval span in cents. The vertical axis represents the synaptic weights (the consonance measure) in arbitrary units. For this graphic, the magnitude of the tonic is artificially lowered, so the rest of the intervals are noticeable.

Table 1 shows the intervals that emerged only after the first sweep. Here we notice additional favored intervals with negative interval span (negative cents). By looking at the intervals that emerged in the octave lower to the tonic, we notice the exact mirror-symmetrical results in the octave of the tonic. In the extended table in the results (see Supplemental Information package, document RESULTS.xlsx, sheet INVERSIONS), we notice the same symmetry even after all the sweeps are performed (Fig. 4).

**Table 1:** Intervals emerged after the end of the first sweep.

Complex sound #		Harmonics emerged in sweep 1 for selected tonic *f*_*0*_—single repetition for each tone									
		Harmonic 1		Harmonic 2		Harmonic 3		Harmonic 4		Harmonic 5	
1	Tone frequency [fraction of *f*_*0*_]	1/5		2/5		3/5		4/5		**5/5**	
	Interval *f*_*0*_ to *f* [cents]	−2786	–	−1586	–	−884	Major 6th	−386	Major 3rd	**0**	**Unison**
2	Tone frequency [fraction of *f*_*0*_]	1/4		2/4		3/4		**4/4**		**5/4**	
	Interval *f*_*0*_ to *f* [cents]	−2400	–	−1200	–	−498	Perfect 4th	**0**	**Unison**	**386**	**Major 3rd**
3	Tone frequency [fraction of *f*_*0*_]	1/3		2/3		**3/3**		**4/3**		**5/3**	
	Interval *f*_*0*_ to *f* [cents]	−1902	–	−702	Perfect 5th	**0**	**Unison**	**498**	**Perfect 4th**	**884**	**Major 6th**
4	Tone frequency [fraction of *f*_*0*_]	1/2		**2/2**		**3/2**		**4/2**		5/2	
	Interval *f*_*0*_ to *f* [cents]	−1200	–	**0**	**Unison**	**702**	**Perfect 5th**	**1200**	**Octave**	1586	–
5	Tone frequency [fraction of *f*_*0*_]	**1**		**2**		3		4		5	
	Interval *f*_*0*_ to *f* [cents]	**0**	**Unison**	**1200**	**Octave**	1902	–	2400	–	2786	–

Only for this explanation, we consider that each sound was presented only once (no repetitions)—this avoids the effects of the synaptic weights adjustments to the obtained results, causing both our- and the Terhardt’s method to predict the same intervals and with the same magnitude of synaptic weights (as a measure for consonance). The intervals with a dark background and white text are in the scope of the octave of interest (where the tonic *f*_*0*_ is the base tone). The intervals that emerged in the lower octave (where the tonic *f*_*0*_ is the tone with the highest frequency) are presented with negative cents. The intervals below the lower octave and above the tonic’s octave are grayed-out.

## Discussion

Here we explain in detail how the neural network succeeds in predicting the emerged intervals and discuss the results' meaning and significance.

**Figure 5:**
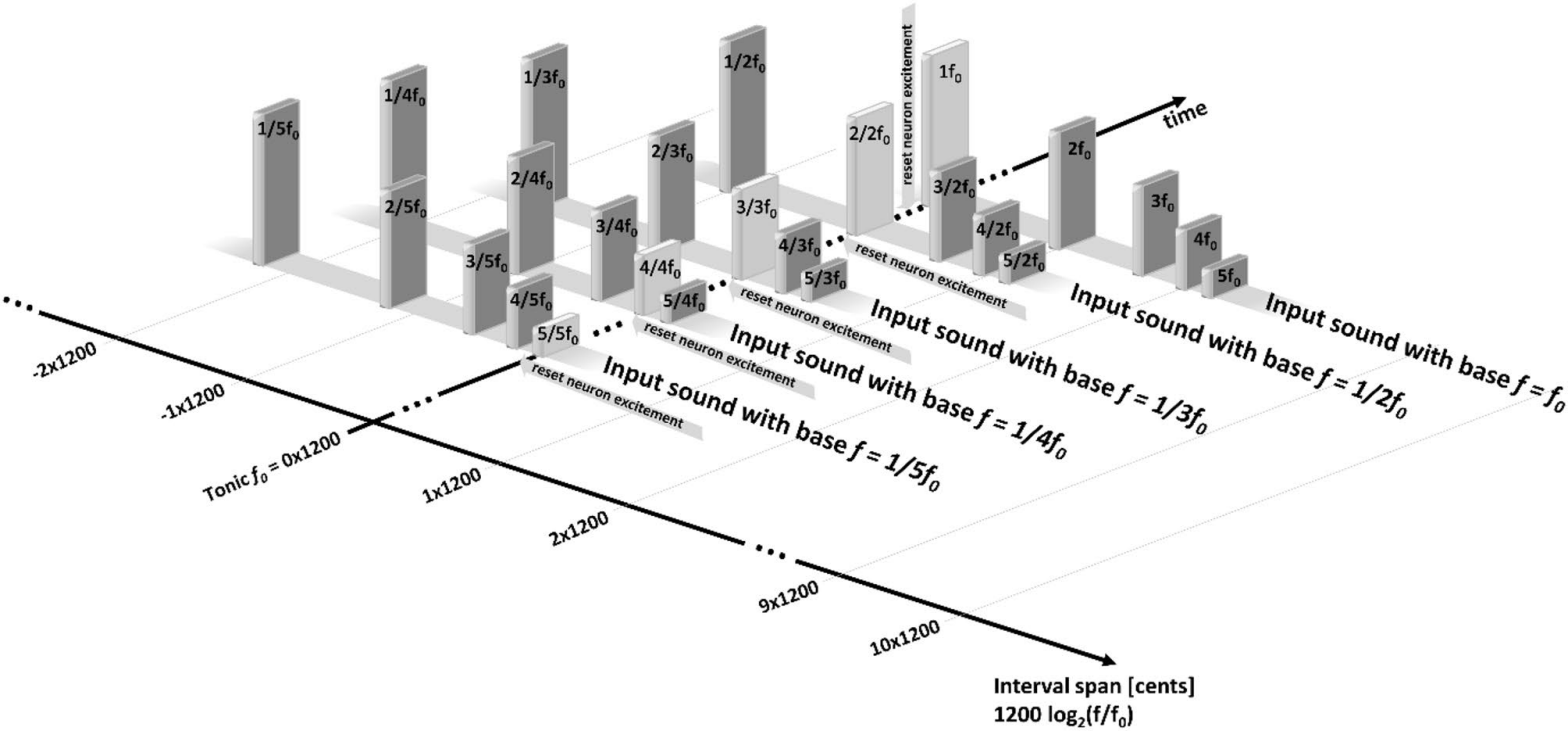
Explanatory snapshot presenting all the complex input sounds containing a selected tonic *f*_*0*_. The vertical 3D bars represent the harmonics within the complex sounds, with their height signifying the harmonic magnitude. The bars of the tonic are darker than those representing the other harmonics. In the image, we note the “reset neuron excitement” arrows, symbolizing that the said value is reset between the presentation of a given sound and its successor.

In Fig. 5, we show all the complex input sounds (including their harmonics) that contain a given frequency *f*_*0*_ (the selected tonic). For simplicity of this explanation, we assume a single repetition of each sound within the first sweep. We start reviewing the events at the moment of the presentation of the sound with a base frequency of 1/5 of *f*_*0*_—that sound contains *f*_*0*_ as its highest (fifth) harmonic. The presentation of the said sound will excite the neural correlates of the harmonics included in the sound. In the next step, as a consequence of the Hebbian learning paradigm (“neurons that fire together, wire together”), the neural correlates of each harmonic from a presented input sound will increase the synaptic weights of their links to the correlates of all the other harmonics within that sound. Since our selected tonic *f*_*0*_ is the fifth harmonic in the sound (with the highest frequency), the intervals that emerge are presented in Table 1 in the row “Input sound #: 1” (the unison is always present due to the design of the model).

After the first sounds, the model resets the neural excitation values to avoid inter-sound harmonic interactions. This way, we lessen the relevance of the input sounds' order to the results. In our previous study^11^, we show that the obtained results are the same regardless of the increasing or decreasing direction of the sweeps. Also, in the said study, we show that the results are not changed if we use a quasi-random uniformly distributed presentation of the input sounds). In the next step, we present the second sound to the neural network—the model strengthens the synaptic weights of the intervals formed by the contained harmonics—the same as for the first sound. Table 1 shows the emerged intervals in the row “Input sound #: 2”. We continue presenting the following three sounds, and finally, we get all the intervals enlisted in Table 1. As per the selected Hebbian-learning rule, the magnitudes of the adjusted synapses will be strongest for the harmonics with the largest magnitudes in the input sound.

In Table 1, we notice a perfect symetry in the emerged intervals in the octave just below- and above the selected tonic *f*_*0*_. Also, the intervals favored in the lower octave contain tones that with the *f*_*0*_/2 form the inversions of the intervals in the tonic’s octave. To explain this appearance, we consider Fig. 4 containing both the lower- and the tonic’s octave after the completed training of 8 sweeps with repetitions ends. The graph in Fig. 4 directly shows that the major third interval (C4–E4) has almost the same consonance magnitude as the major third interval G3#–C4. That is expected since they are both major third intervals, yet in different keys. Although the graph shows that the tone G3# forms the minor sixth interval C3–G3# (the minor sixth is an inversion of the major third) may raise appearance about the equivalence of the intervals with their inversion, that is misleading—the example interval shown on the left portion of the graph with almost equal consonance as C4–E4 is the major third in the key of G3#. The interval inversions may start making sense in this context only if we consider the octave equivalency of the tonic C4 with C3 (so we “get” the minor sixth C3–G3#), but we aim to show what the model yields the results without the help of the octave equivalency.

The known methods of forming musical scales require the octave equivalence principle; without it, they cannot “contain” the tones of the scale in the scope of a single octave. In contrast, our model *does not use the octave equivalence* (not explicitly nor implicitly)—the intervals emerge “naturally” (“organically”) from the inner workings of the model. However, the octave equivalence is evident in the results (the most favored, meaning most consonant interval, after the unison is the octave).

The difference between our- and Terhardt’s models is the dependency on the model's history. In the upper example of the first sweep, we used a single repetition per input sound to avoid the effect of synaptic weights even on our model results. In the subsequent sweeps, the same set of 5 sounds will continue to strengthen the synaptic weights (the consonance magnitude) of the emerged intervals, and as we see in the results, these intervals will end up being the most consonant for both, Terhardt’s- and our model. Our previous paper^11^ named these intervals as *First-order partners*. Those are also the most consonant intervals, as per Helmholtz^8^.

We continue to analyze the events when we present the same five complex sounds in the second sweep. For this sweep, we use more than one repetition for each sound, as our model truly is designed, which allows for the current synaptic weights to affect the excitation of the neural correlates, which (through the Hebbian learning paradigm) affect the adjustments of the synaptic weights in the subsequent repetitions and sweeps. Here is how: once the first sound from Fig. 5 is presented for the first time in the second sweep, each neural correlate of the included harmonics is excited—five in total. Each of these five excited neural correlates, in the second repetition of the same sound, will pass a portion of their excitements to the neural correlates of their including intervals that emerged in the first sweep (as per Table 1)—through the links whose synaptic weights got increased in that first sweep. Since in the first sweep, each of the five harmonics (neural correlates) becomes linked to four other tones, in the second presentation of the second sweep, a total of 5∙4 = 20 tones will be (additionally) excited—simultaneously. Since all these tones are excited simultaneously, the Hebbian-learning feature will increase the synaptic weights between them, increasing their consonance magnitudes of the intervals that all these 20 tones form. When we present the rest of the four complex sounds to the neural network, the neural correlate of the selected tonic will gain many more interlinked neural correlates, creating newly emerged intervals of the said tonic—yet with smaller magnitude than the *First-order partners*. Among those (large number of) newly emerged intervals, we find the minor second, major second, minor third, tritone, minor sixth, minor seventh, major seventh—and many more. Our previous paper^11^ names these intervals as *Second-order partners*.

**Figure 6:**
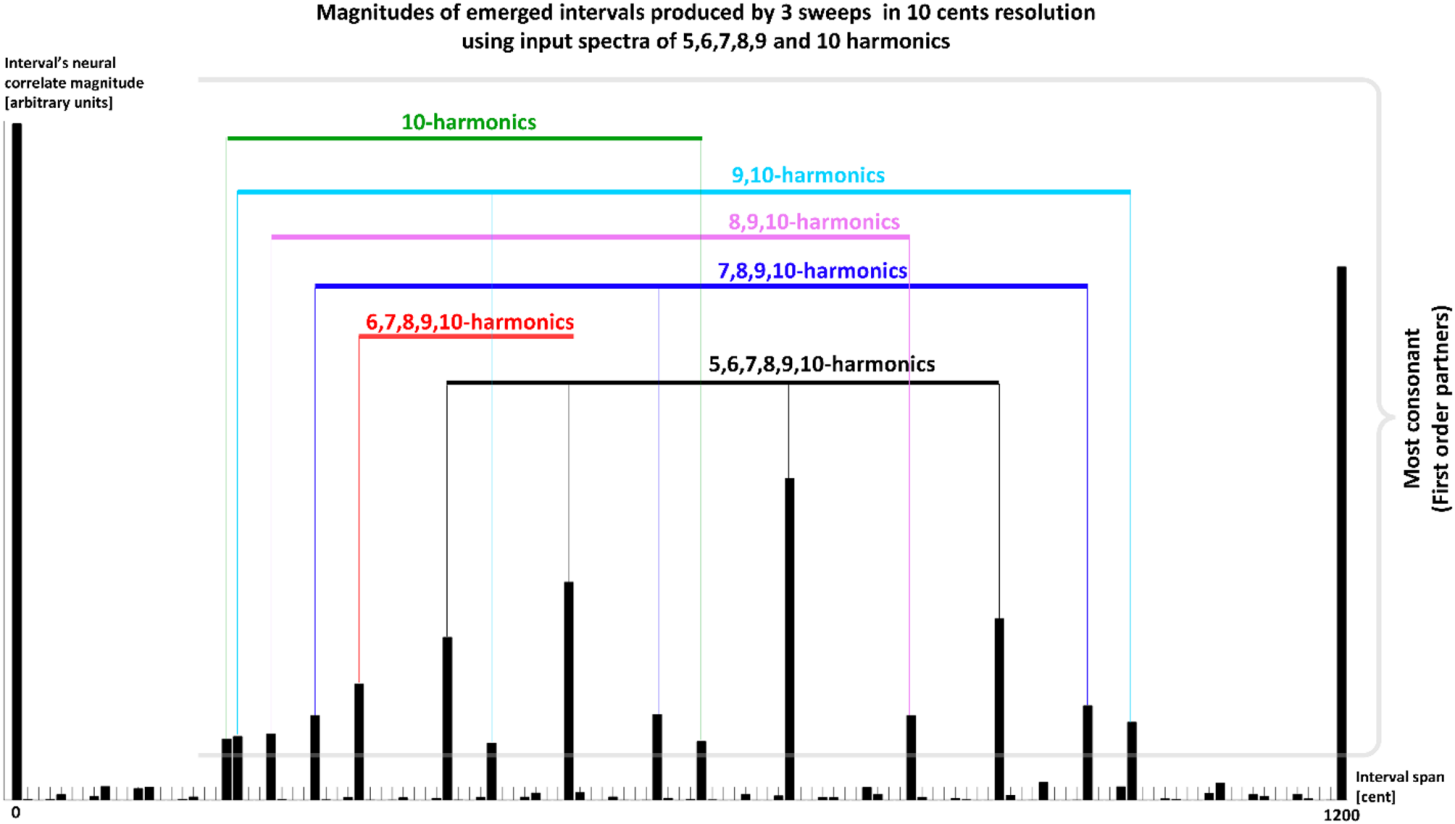
Comparative presentation of the most-consonant emerged intervals when using input sound of 5, 6, 7, 8, 9, and 10 harmonics. We notice that input spectra of more than five harmonics reinforce and produce the *most consonant intervals* that emerged from the spectra of the lower number of harmonics, keeping the most consonant and adding their favorite specific intervals. For comparison, we show the position of the 12 most consonant intervals that emerged from the 5-harmonics input spectra below the horizontal axis. The upper graphs are produced using a coarse input frequency resolution of 10 cents, and for each number of harmonics, we performed three sweeps.

In our study, we review complex input sounds made of five harmonics, so it is expected that the emerged intervals would be members of the 5-limit tonal system. If we use complex input sounds with six harmonics, again in decreasing order of magnitude when the frequency increases, the emerged intervals will contain the same most-consonant intervals produced from the 5-harmonics sound, with additional intervals specific to the 6th harmonic (6*f*_*0*_). It is worth noting that the 6 is a multiple of 2 and 3; therefore, it should be already part of the 5-limit system. That is true, although due to the design of our model, when using 5-harmonics sound, its emerged intervals have low synaptic weights, and therefore they are not noticeable. The same pattern repeats with any number of additional harmonics. We test these situations with a significantly coarser frequency resolution of 10 cents. Comprehensive simulations of the situations with an increased number of harmonics and different harmonics mangitude signature (not only in decreasing order of frequency), using 1 cent frequency resolution, will take an unreasonably long calculation time with the available computational power to which we have access. A comparative result of the most-consonant intervals emerged with the input sounds ranging from 5 to 10 harmonics is presented in Fig. 6. This result and the comprehensive analysis with a very coarse resolution of 100 cents used in our previous study^11^ show that the order of consonance of the emerged intervals is significantly affected by the selected number of harmonics and their magnitude distribution (the experience, exposure to various auditory contexts, the familiarity). The result that familiarity is a predictor of consonance agrees with recent experimental results by multiple authors—as in Eerola and Lahdelma^19^, reporting that the variance in consonance ratings comes 46.2% from familiarity (experience) and only 19.3% from roughness/harmonicity. This result suggests why our model does not perfectly predict Helmholtz’s consonance order of the least-consonant intervals: our selected corpus of input sounds (our model’s auditory “experience”) differs from the lifelong auditory exposures of the participants' in Helmholtz’s consonance studies. Nevertheless, considering the whole population of 1200 intervals our model yields (after a sufficient number of performed sweeps), we are satisfied with this partial matching—which supports familiarity as a predictor of consonance.

### Terhardt’s model

Interestingly, in^23^, Terhardt was very close to achieving the same results as our present study. Terhardn’t model demonstrates the causes for the intervals’ discreteness in the musical scales (familiarity). Still, he did not succeed entirely only because the strengths (weights) of the changing associations between the harmonics correlates in his model have no effect on his system’s future behavior. That is understandable because he does not model a neural network but a statistical (“mechanical,” non-biological) apparatus that calculates the frequencies of harmonics’ incidences in the input stimuli. Interestingly, Terhardt’s model also does not require implementing the octave equivalency to produce its results. More in-depth analyses of the processes within these two models reveal that the missing feedback loop in Therhardt’s model—see Eqs. (4) and (4a) in the Algorithm.pdf file in the Supplementary Material package—causes it to yield only six intervals; those are the top-most consonant intervals—which corresponds to the First-Order Partners as per^8^ and^11^. That model does not yield any other interval, not even with a small magnitude. On the other hand, the existence of such a feedback loop in our model (the changing Hebbian synaptic weights affect the future behavior of the model) allows for the emergence of the Second-Order Partners (as per our previous study^11^), which adds additional intervals from the 5-limit system and (with faint magnitudes) all the rest of the 1200 intervals.

## Conclusion

**Figure 7:**
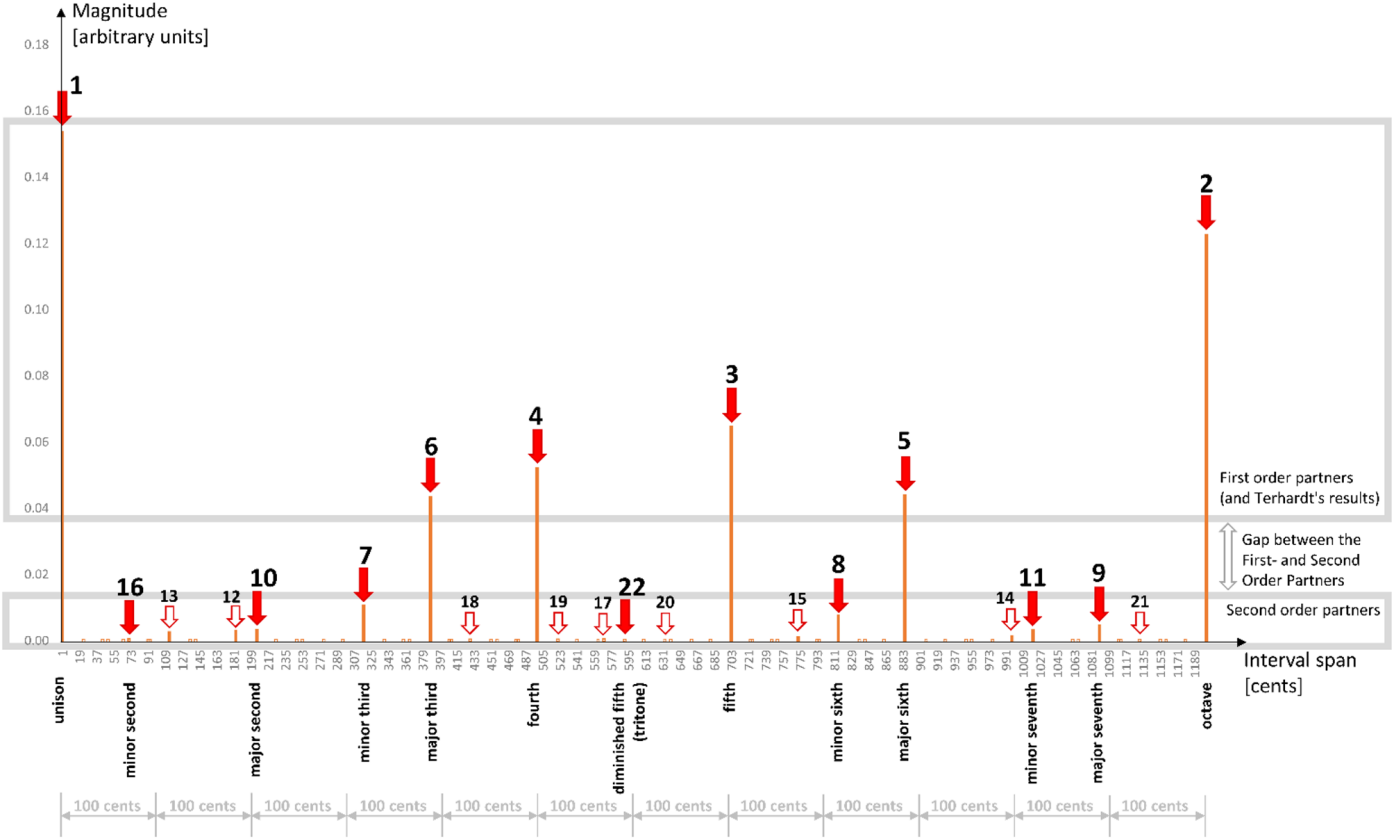
Graphical representation of the obtained comparison results. Sourced from Fig. 3, this figure shows a bar graph of magnitudes of the emerged intervals against the interval span dimension. The graph clearly resembles Fig. 2, demonstrating the discrete nature of the emerged intervals and the approximate invariance of that discreteness on the used input frequency resolution. Also, compared to Fig. 2, one can observe that the consonance pattern of the most consonant intervals is preserved as per our previous study^11^. The number annotations above the arrows show the order of the emerged interval in the list order by decreasing magnitude (lower numbers represent more consonant intervals). The filled arrows point to the matching Helmholtz’s list intervals as per Fig. 3. The empty arrows point to the intervals that our model yielded as more consonant than 16th or 22nd. The upper grayed area contains the First-Order Partners (highly consonant intervals). The lower grayed area contains the Second-Order Partners (less-consonant intervals); the wide gap between the First-Order Partners and Second-Order Partners magnitudes is observable. The intervals that emerged from Terhardt’s model are all in the zone of the First-Order Partners.

Finally, we observe that the processes simulated by our model predict the following results: the discreteness of the tones (Fig. 7), the size of the smallest emerged most-favored interval span for the 5-harmonic input sounds of approximately one semitone (Fig. 7), the yielding of 5-limit Just Intonation intervals as most-preferred (most consonant)—reproducing the upper portion of the Helmholtz’s list of tonal intervals ordered by consonance (Fig. 3), the consonance symmetry around the tonic (Fig. 4). To avoid confusion, we should clarify that the resuilts demonstrate that biologial auditory systems we modeled favor the 5-limit Just Intonation scale, however this scale is not practical to be used in musical performace and it did not play significant role in Western music. In the lower part of the consonance-ordered list, our model yields tones that with their adjacently yielded tones form much-smaller-than-100-cent intervals; yet, those faint intervals could be considered more each other's alternatives than adjacent in the emerged list (for example, the greater and the lesser major second). The invariance of the favored intervals on frequency resolution is also evident; that invariance cannot be pronounced “perfect” for input resolutions coarser than the expected emerged intervals. Our model produces the said results without using the octave equivalence enabled mapping across octaves—the results emerge within a scope of the octave “naturally” (“organically”) from the inner workings of the model. In addition, we do not refute the possibility that the roughness (or other causes) affects the subjective feeling of consonance. Still, we highlight that our model produced the results without considering those other causes; we offer citations of EEG studies relating the familiarity with the consonance and dissonance phenomena which helps bridge the gap between the magnitude of the synaptic weights and the evoked subjective sensations. In conclusion, by having the Hebbian-learning paradigm and harmonicity as the main and only significant features of our model, in the scope of obtained results, we propose that these two features are sufficient (although not necessarily required) for any system to yield the said. Therefore, the Hebbian learning and harmonicity should play crucial roles for biological systems in processing pitch (contributing to arguments that “place” the “pitch processor” in the brain), sensations of consonance, consequently—music perception in general.

## Methods

In this study, we reuse our previous model initially disclosed in^11^. Although the details and the source code are well documented in the said paper, for readability, here we describe the model’s core features (Fig. 1). Also, the Introduction section of this new paper contains definitions and essential explanations of sound and its basic properties, the continuity of the sound spectrum, the “central pitch processor,” neural networks, Hebbian learning paradigm (neuro-plasticity), neural correlates, the just intonation-, the Pythagorean- and the well-tempered tunning systems.

Our model consists of a simple 2-layer feed-forward neural network featuring Hebbian learning between the first and the second layer, meaning, in case of an incidence of a neuron of the first layer and a neuron of the second layer firing simultaneously, the weight of the first-layer neuron’s synapse toward the second-layer neuron will increase. The neural network simulates the first few layers of the auditory neural system featuring Hebbian learning. Each neuron from each layer represents a neural correlate to a specific tone of the auditory spectrum made of 12,000 frequencies, where two adjacent tones have a frequency distance of 1 cent = 1200log_2_(*f*_*n*+1_/*f*_*n*_), where *f*_*n*+1_ and *f*_*n*_ are tones’ frequencies in Hz. The lowest tone in the spectrum is C0 (16.35159783 Hz), and the highest is C10 (16,744.03618000 Hz). The neurons are placed in tonotopic order by the tones whose correlate they represent; the tonotopy is not required to produce the results, but it is beneficial to facilitate the analyses. The *n*th layer-1 neuron has a static innate excitatory synaptic association to the *n*th layer-2 neuron. All the synaptic associations between the neurons of a different order (index) are of the Hebbian type. Formally, for given *i*th neuron from the layer-1 and *j*th neuron of the layer-2, their synaptic associations are innate-static when *i* = *j* and Hebbian/changing for *i ≠ j*. The chose of using a single neuron per frequency band in the model is not a requirement for the model to produce the results—the results would be produced even if we use a group of neurons as a correlate per frequency band. Still, by using a single neuron per frequency band we demonstrate the minimal neural model that is still able to produce the results. The use of a single neuron per frequency band is also supported by the tonotopy/cochleotopy—not so much about the inter-neuron relative spatial position but more about the discriminative correlation of the neuronal correlates per frequency band. The tonotopy/cochleotopy is a well-researched and agreed upon feature of the auditory brain systems—both the base and the deeper neural structures in the brain^25–27^. Even more, all the modality in the brain feature such perceptual maps. As per Hackett, "Functionally, the representation of frequency is the most robust topographically distributed physiologic feature known in auditory cortex. The presence of these maps indicates that auditory processing within each area is spectrally distributed, such that the interactions between auditory areas appear to be shaped by tonotopic organization. For example, connections between matching isofrequency domains in different areas are stronger than connections between unmatched locations."^25^. The finding that the iso-frequency areas exibit strongest connections are supporting our design of maintaining strongest iso-frequency connections between the two neuronal layers in the model.

The neural network is fed with a train of sound stimuli (Fig. 1). Each sound consists of a base tone with a given frequency *f* from the auditory spectrum described above and four harmonics in descending order of amplitudes: 2*f*., 3*f*., 4*f*., and 5*f*. The train sweeps the said auditory spectrum from the lowest to the highest frequency while the neural network is learning. With each stimulus, some of the Hebbian synaptic weights are adjusted, and they influence the future behavior of the network. The choice of this specific harmonic signature of complex sounds (a base tone plus four harmonics, multiples of the base tone frequency, in descending order of amplitudes) is made according to the findings^11^. In that study, we test all the possible spectral amplitude signatures with up to 9 spectral components within the selected (high) precision, and we demonstrate that only this type of spectrum (five harmonics in decreasing order of magnitudes) reproduces Helmholtz’s consonance ordered list perfectly^8^, considering the caveat that in our previous study we use well-tempered scale different than Helmholtz. In that previous paper, we demonstrate that the resultant pattern is the same regardless of the direction of the sweep—ascending, descending, or in a quasi-random order of uniformly distributed sounds.

Due to length restrictions, we describe the algorithm of the neural network simulation in the document Algorithm.pdf, found in the Supplementary Information package, together with the complete MATLAB program code modeling the system in the file InputPerSemitone_100.m.

A similar approach is used by Terhardt^23^, where the author feeds his model with speech sounds. In our investigation of Terhardt’s model, we use the same input stimuli as our model, and we repeat the same process to have a fair comparison. However, as mentioned earlier, our model differs from Terhardt’s in the model’s behavior depending on the current synaptic weights—in Terhardt’s model, there is no such a dependency. The full MATLAB script modeling the system of^23^ is located in the Supplementary Information package, as a file TERHARDT_InputPerSemitone_100.m.

The calculations were performed on a personal computer running the Microsoft Windows 10 operating system. The processing unit consisted of 4 physical cores/8 logical processors (Intel i7-6700HQ, at 2.6 GHz). All the computations lasted 22 × 24 h.

We collected data about various musical scales used across ages and cultures^20,28–33^. The data is of diverse precision, naming and notation. The collected intervals are enlisted with the base tone C4 with a frequency of 261.6255653 Hz, their interval span in cents, and an indicator showing whether the interval is a member of the Just Intonation scale as per^34^. There are 1162 intervals in the full list, which can be found in the RESULTS.xlsx document located in the Supplemental Information package—the first three columns titled “Collected Scale Members” in the sheet named “Comparison.” Note that the resolution/precision of the frequency span for some of the presented intervals goes below 1 cent.

We review the results in a scope of a single octave from the middle of the auditory spectrum; we have selected the C4–C5 octave to avoid the apparent boundary influences near the edges of the auditory spectrum.

The MATLAB model we used produced a “.mat” data file containing a matrix of the Hebbian synaptic weights between the neural correlates of the model’s first and second layer. The matrix is of dimension 12,000 × 12,000—yet, here we are only interested in the synaptic weights between the tones of the C4–C5 octave, more precisely, the synaptic weights that the tone C4 (the selected tonic) relates to the tones of the same octave. That gives us a 1 × 1200 vector (a single-dimensional list), one magnitude value for each synaptic weight from the tonic toward each tone (the target) of the same octave—each value representing the magnitude related to interval the tonic forms with the target tone (column “Magnitudes” in Fig. 3). In other words, the first value of the list represents the magnitude (of the synaptic weight) of the interval with a span of 0 cents, the second value—the magnitude of the interval with a span of 1 cent, continuing up to the octave interval spanning 1200 cents. In this list, we add a column filled with the interval span values (column “Emerged interval span” in Fig. 3). We order this list by the obtained synaptic weight magnitude value in descending order, and we add a third column, enumerating each interval with values from 1 to 1200, where we give the order of one to the highest-, and the order of 1200 to the lowest magnitude interval (column “Decreasing order of magnitude” in Fig. 3).

Finally, we merge the “Helmholtz’s intervals” table with the “Intervals emerged from our model” table in Fig. 3 to visually demonstrate the most-preferred intervals yielded from our model in relation to Helmholtz’s list of intervals ordered by consonance.

Since our model yielded all the possible intervals from the auditory spectrum (after performing a sufficient number of sweeps), we see no use in comparing the (yielded) intervals with the 1162 intervals collected from various literature sources.

## Supplementary Information

Supplementary Information 1.

Supplementary Information 2.

Supplementary Information 3.

Supplementary Information 4.

Supplementary Information 5.

Supplementary Information 6.

Supplementary Information 7.

## Data Availability

The online version contains all the data and MATLAB source code in the supplementary material file.
