# Supplementary material for: Nonspecific hebbian neural network model predicts musical scales discreteness and just intonation without using octave-equivalency mapping
Source: Sci Rep. 2022 May 25;12:8795. doi: 10.1038/s41598-022-12922-x (PMC9132910; doi:10.1038/s41598-022-12922-x)
Supplement: Supplementary file 1 — Supplementary Information 1. [file 41598_2022_12922_MOESM1_ESM.pdf]

The Consonance-Emerging Hebbian-Learning Generic Neural Network Model Predicts Discreteness of Musical Scales and Just Intonation Scales without Using Octave-Equivalency Mapping

Algorithm

Here we show the high-level outline of the algorithm implemented in the MATLAB program code that simulates the model. Since both our previous and current studies uses similar model, the following description matches both studyes, and the improvements from our previous study are annotated.

The neural network, comprises of two layers of neurons named first layer and second layer. Both layers have the same number of neurons (neural correlates) equal to the total number of harmonics that can input in the neural network. The neurons of the first layer are fed with harmonic input sounds in a tonotopic manner: the neuron with index i from the first layer is connected with an innate and static synaptic connection to the neuron with the same index of the second layer, where $i = 1, \dots, N$, having:

N = 12000 (120 in our previous study [11]) (1)

Consequently, N represents the number of frequency bands the spectrum analyzer is breaking down the input auditory signal. Because of the tonotopy the same number represents the number of neurons within each of the first and second layer of the neural network.

For simplicity, instead of referring to these neurons as neural correlates of the given frequency band, say the neuron from the first layer correlated to the band with a middle frequency of C in the octave 4, we are using the simplified denotation the first layer neuron C4. The synaptic links departing from the first layer toward the second layer are innately created with identical weights, but within the training phase, over time, their weights change according to the Hebbian-learning rules. The complete rules that define the neural network are explained in what follows.

The activity rule for first layer of the neural network is given with the following equations:

$$\boldsymbol{PSP_1(t+1) = I(t+1)} \quad (2)$$

$$\boldsymbol{PSP_1(t+1) = I(t+1) + Axon_2(t)} \quad (3)$$

where

(2) gives the rule of the feed-forward version of the neural network

(3) gives the rule of the recurrent version of the neural network (only for our previous study, the recurrent version is not analyzed in this study)

$\boldsymbol{PSP_1}$ is the column vector of post-synaptic potential (excitement) magnitudes of the neurons in the first layer. The dimensions of the vector $\boldsymbol{PSP_1}$ are $1xN$, where N is given by (1). Consequently, with $PSP_{1,i}$ we denote the post-synaptic potential of the ith neuron within the first layer.

$\boldsymbol{I}$ is the column vector of neural signal magnitudes coming from the non-linear transducer/spectrum analyzer (an auditory model of the environment + the cochlea). With I_i we denote the signal strength of the ith output from the non-linear transducer/spectrum analyzer.

$\boldsymbol{Axon_2}$ is the column vector of the output magnitudes (the signal magnitudes traveling through the links between the neurons) from the second layer. With $Axon_{2,i}$ we denote the output magnitude of the ith neuron within the second layer.

$(\boldsymbol{t})$ and $(t+1)$ denote the values of the variables in the discrete times (ticks) t and $t+1$.

The activity rule for second layer of the neural network is given with the following equations:

$$\boldsymbol{PSP_2(t+1) = W(t) \times Axon_1(t)} \quad \text{(our model, having a feedback element)} \quad (4)$$

$$\boldsymbol{PSP_2(t+1) = Axon_1(t)} \quad \text{(Terhardt's model [22], not having a feedback element)} \quad (4a)$$

where

$\boldsymbol{PSP_2}$ is the column vector of post-synaptic potential magnitudes of the neurons in the second layer. The dimensions of the vector $\boldsymbol{PSP_2}$ are $1xN$, where N is given by (1). Consequently, with $PSP_{2,i}$ we denote the post-synaptic potential of the ith neuron within the second layer.

$\boldsymbol{W}$ is the matrix of the changeable synaptic weights of the neural connections that depart from the first layer toward the second layer. With $W_{i,k}$ we denote the synaptic weight of the neural connection departing from the ith neuron of the first layer toward the kth neuron of the second layer.

The values of the synaptic weights are innately set according to the following rule:

$$W_{i,k} = \begin{cases} 0, & i \neq k \\ 1, & i = k \end{cases} \tag{5}$$

$\boldsymbol{Axon_1}$ is the column vector of the output magnitudes from the first layer. With $Axon_{1,i}$ we denote the output magnitude of the ith neuron within the first layer.

The activation rule of a single neuron within any layer is given with the simplest possible rules:

$$\boldsymbol{Axon_1}(\boldsymbol{t}+\boldsymbol{1}) = \boldsymbol{PSP_1}(\boldsymbol{t}+\boldsymbol{1}) \tag{6}$$

$$\boldsymbol{Axon_2}(\boldsymbol{t}+\boldsymbol{1}) = \boldsymbol{PSP_2}(\boldsymbol{t}+\boldsymbol{1}) \tag{7}$$

The Hebbian learning rule is given with the following equation:

$$W_{i,k}(t+1) = W_{i,k}(t) + r \times Axon_{1,i}(t+1) \times Axon_{2,k}(t+1) \tag{8}$$

where

r is the learning speed factor.

In what follows we disclose the neural network learning algorithm.

Step 1: Set the values of the synapses W according to (5).

Step 2: Set $\boldsymbol{PSP}$ and $\boldsymbol{Axon}$ values to zeros.

Step 3: Set $i = 1$. The value of i denotes the index of the frequency band within the tonotopic order of the neural correlates.

Step 4: Present to the system an input tone with frequency f_i which correlates to the frequency band i. By the design of the sub-system 1, its outputs are given by the input column vector $\boldsymbol{I}$. This vector has non-zero descending values for I_i, I_j, I_k, I_l, I_m, where i, j, k, l and m are the indexes of the neural signals in $\boldsymbol{I}$, correlated with frequencies f_i, $2f_i$, $3f_i$, $4f_i$ and $5f_i$ respectively. All other elements of the column vector $\boldsymbol{I}$ are set to zeros. We shall refer to the input column vector $\boldsymbol{I}$ as Input Set.

Step 5: Apply the activity rules of the first and second neural network layers, provided by (2) and (4); for Therhardt's model use (4a) instead of (4).

Step 6: Apply the activation rules of the first and second neural network layers, provided by (6) and (7).

Step 7: Apply the Hebbian learning rule given by (8).

Step 8: Repeat steps 4 to 7, R number of times ($R = 10$). We shall denote the parameter R with the name Input Set Repetitions. After the steps 4 to 7 have been repeated R number of times, proceed to step 9.

Step 9: Increment the value of i by one to use the next complex sound from the input auditory spectrum. Until i becomes bigger than N, given by (1), proceed with step 1, otherwise proceed with step 9. This step effectively changes the frequency of the input pure tone to the next tone in the input train. This causes the input tone frequency to sweep the whole 10 octave auditory spectrum, repeating the presentation of each input tone to the system for R consecutive number of times.

Step 10: Repeat the steps 2 to 9, S number of times ($S = 8$). We shall denote the parameter S with the name Training Series (sweeps). After the steps 2 to 9 have been repeated S number of times, proceed to step 11.

Step 11: Stop.

Each sound stimulus is presented to the model ten times in a row, and then before presenting the next sound, the post-synaptic potential of the neurons is reset to zero, so there is no "short-term memory" inter-sound interference; with this we avoid inter-sound harmonic polution based on the order of presentation of the tones (achieving presentation order invariance). Thus, previously presented sounds affect the "perception" of the subsequent sounds only through the adjusted Hebbian synaptic weights. After performing ten sweeps of the full auditory spectrum, the process ends.

In the following figure we show the flow-diagram of the algorithm, with annotated "STEPs" from above.

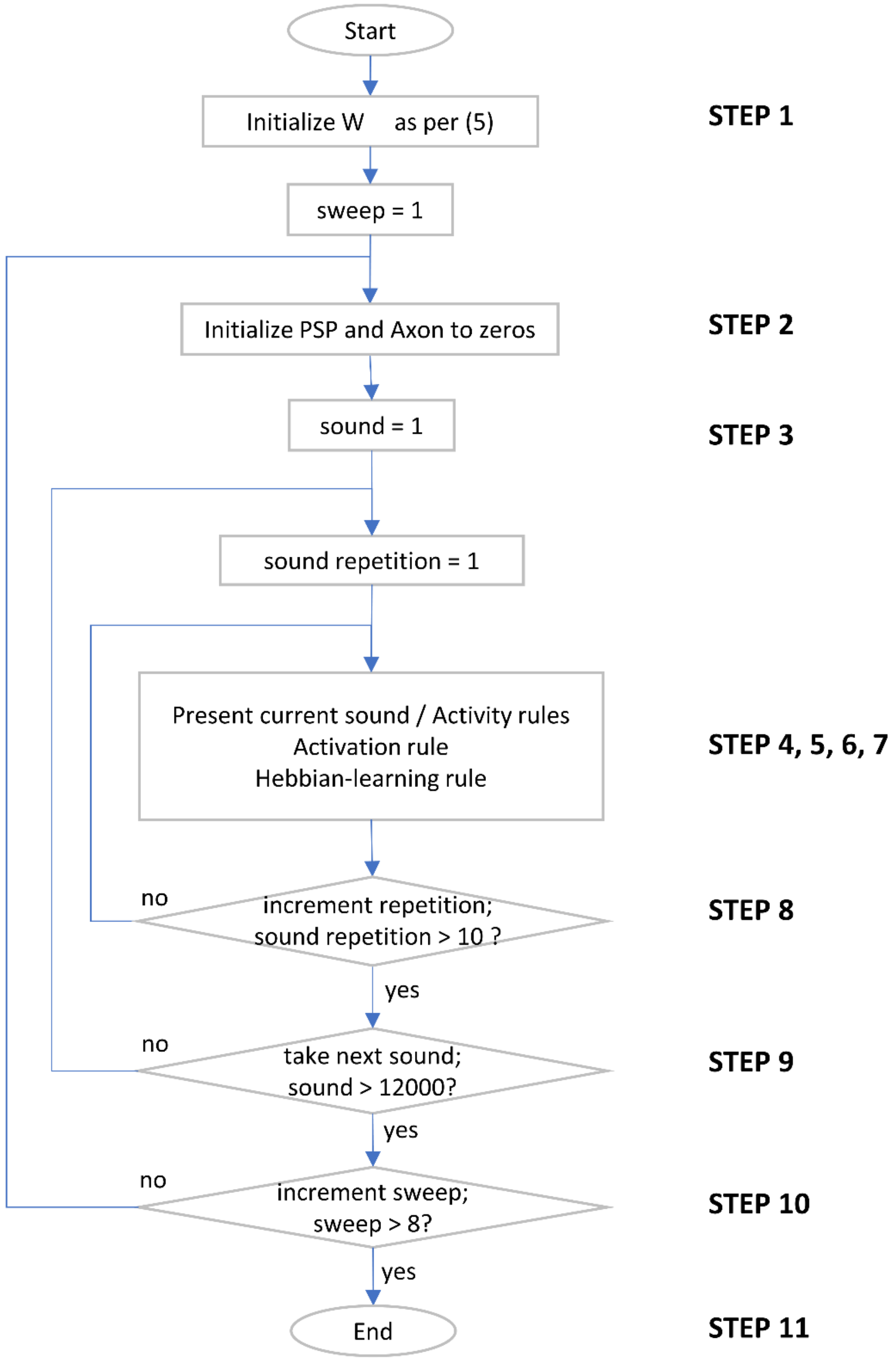
Start
Initialize W as per (5)
STEP 1
sweep = 1
Initialize PSP and Axon to zeros
STEP 2
sound = 1
STEP 3
sound repetition = 1
Present current sound / Activity rules
Activation rule
Hebbian-learning rule
STEP 4, 5, 6, 7
no
increment repetition;
sound repetition > 10 ?
STEP 8
yes
no
take next sound;
sound > 12000?
STEP 9
yes
no
increment sweep;
sweep > 8?
STEP 10
yes
End
STEP 11
